# Is COVID-19 Infection a Multiorganic Disease? Focus on Extrapulmonary Involvement of SARS-CoV-2

**DOI:** 10.3390/jcm13051397

**Published:** 2024-02-28

**Authors:** Gauthier Duloquin, Thibaut Pommier, Marjolaine Georges, Maurice Giroud, Charles Guenancia, Yannick Béjot, Gabriel Laurent, Claudio Rabec

**Affiliations:** 1 Department of Neurology, CHU Dijon-Bourgogne, 21000 Dijon, France; gauthier.duloquin@chu-dijon.fr (G.D.); maurice.giroud-ext@chu-dijon.fr (M.G.); yannick.bejot@chu-dijon.fr (Y.B.); 2 Laboratory of Cerebro-Vascular Pathophysiology and Epidemiology (PEC2) EA 7460, University of Bourgogne, 21000 Dijon, France; thibaut.pommier@chu-dijon.fr (T.P.); charles.guenancia@chu-dijon.fr (C.G.); gabriel.laurent@chu-dijon.fr (G.L.); 3 Department of Cardiology, University Hospital of Dijon, 21000 Dijon, France; 4 Department of Pneumology and Intensive Care Unit, Reference Centre for Rare Lung Diseases, Dijon University Hospital, 14 Boulevard Gaffarel, 21000 Dijon, France; marjolaine.georges@chu-dijon.fr; 5 Centre des Sciences du Goût et de l’Alimentation, INRA, UMR 6265 CNRS 1234, University of Bourgogne Franche-Comté, 21000 Dijon, France

**Keywords:** SARS-CoV-2, multiorganic, COVID-19 infection

## Abstract

First described in December 2019 in Wuhan (China), COVID-19 disease rapidly spread worldwide, constituting the biggest pandemic in the last 100 years. Even if SARS-CoV-2, the agent responsible for COVID-19, is mainly associated with pulmonary injury, evidence is growing that this virus can affect many organs, including the heart and vascular endothelial cells, and cause haemostasis, CNS, and kidney and gastrointestinal tract abnormalities that can impact in the disease course and prognosis. In fact, COVID-19 may affect almost all the organs. Hence, SARS-CoV-2 is essentially a systemic infection that can present a large number of clinical manifestations, and it is variable in distribution and severity, which means it is potentially life-threatening. The goal of this comprehensive review paper in the series is to give an overview of non-pulmonary involvement in COVID-19, with a special focus on underlying pathophysiological mechanisms and clinical presentation.

## 1. Introduction

The SARS-CoV-2 virus (Severe Acute Respiratory Syndrome Coronavirus 2 (SARS-CoV-2)), the cause of Coronavirus disease 19 (COVID-19), has turned out to be one of the greatest challenges in modern medicine. First described in December 2019 in Wuhan (China), it rapidly spread worldwide, leading to the declaration of a pandemic in March 2020 [1]. To date, over 700 million cases have been reported, including 6.9 million deaths worldwide (https://www.worldometers.info/coronavirus/, accessed on 3 January 2024).

As a consequence of the rapid spread of the disease, many countries decided to close borders and impose internal quarantines to reduce the spread. The estimated disease-related mortality rate is around 2–5%, according to the community, but it may reach as high as 7% [2].

**Figure 1:**
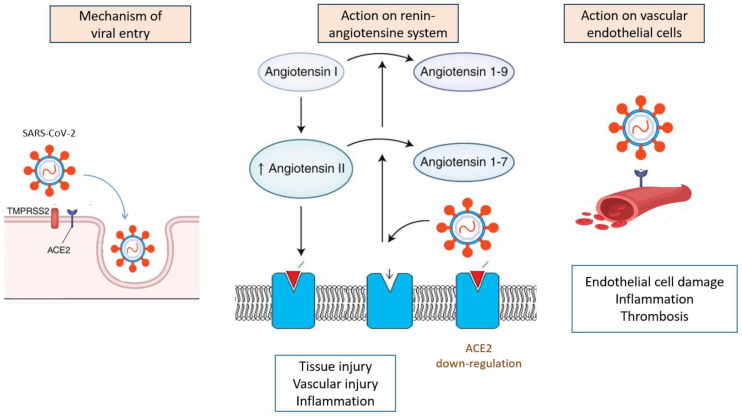
Summary of pathophysiological and molecular mechanisms of SARS-CoV-2 infection.

COVID-19 is known to mainly cause pulmonary diseases. However, evidence is growing that SARS-CoV-2 can affect many organs, including the heart and vascular endothelial cells, and cause haemostasis, CNS, and kidney and gastrointestinal tract abnormalities (Figure 1). In fact, all organs can be affected by the virus [2], and SARS-CoV-19 infection presents a large number of clinical manifestations that can be potentially life-threatening. As a consequence, the pathologic features of COVID-19 are variable in distribution and severity.

The disease presentation mode can largely vary from mild to severe forms. Mild forms are characterized by non-specific symptoms such as fever, vomiting, dysgeusia, and headache with no or mild respiratory symptoms [3]. Knowledge of these extrapulmonary manifestations can help in detecting the mild and moderate forms, which can aid in early diagnosis, and rapid quarantining can prevent community spread. The aim of this paper in the series is to focus on the non-pulmonary involvement in COVID-19. Our goal is to discuss clinical non-pulmonary manifestations with a special focus on the underlying pathophysiology mechanisms of the disease to better understand COVID-19 challenges and give a basis to help management and infection control. The coagulation abnormalities and thromboembolic complications of SARS-CoV-2 infections are addressed in detail in other articles of this series [4].

## 2. Pathophysiological Aspects of SARS-CoV-2 Infection

Even if the mechanisms underlying SARS-CoV-2 are incompletely understood, data suggest a combination of immune dysregulation, the depletion of immune cells, complement activation, and the triggering of the coagulation cascade. Increasing evidence suggests an essential role of vascular endothelium as a critical target for SARS-CoV-2 and the resulting cytokine storm and the main effector for the pro-inflammatory and pro-coagulant status of COVID-19 patients [5]. Pre-existing impaired endothelial function may predispose patients to severe forms of COVID-19. This could explain the fact that conditions characterized by endothelial dysfunction, such as coronary artery disease, hypertension, obesity, and diabetes, are associated with impaired prognosis [6,7].

Both angiotensin-converting enzyme 2 (ACE2) and transmembrane protease serine 2 (TMPRSS2) receptors seem to be the main targets of SARS-CoV-2 [8]. Angiotensin-converting enzyme 2 is not only an enzyme but also a functional receptor on cell surfaces. ACE2 is expressed in many cell types and tissues, including the lungs, heart, blood vessels, kidneys, liver, and gastrointestinal tract. SARS-CoV-2 contains proteins that allow it to bind specifically to ACE2 and, thus, invade the cells responsible for the symptoms. SARS-CoV-2 can also cause renin–angiotensin–aldosterone system activation, which leads to COVID-19 progression, especially in patients with comorbidities such as hypertension, diabetes mellitus, and cardiovascular disease [9].

ACE2 has a critical role in local and systemic haemodynamic and inflammation and is fundamental for vasoregulation, tissue liquid volume maintenance, and electrolyte balance. A link between the expression of ACE2 in different organs and their potential risk during SARS-CoV-2 infection was suggested. High-risk tissues were defined as containing a >1% proportion of ACE2 expression and including the respiratory tract (2%), lung (>1%), heart (>7.5%), ileum (30%), kidney (4%), and bladder (2.4%) [10]. This can explain the higher burden in tissues expressing those receptors and may explain the constellation of symptoms commonly encountered among patients with COVID-19 [8]. In addition, ACE2 tends to be expressed at higher levels in males and increases with age [11]. Finally, it was reported that the level of ACE2 expression could be related to differences in the genetic predisposition to COVID-19. This could explain the lower infection rate in the African population and a higher one in Asians, which is related to differences in ACE2 expression in both populations [12,13].

Additionally, SARS-CoV-2 generates a downregulation of those receptors, decreasing their number, resulting in elevated angiotensin II activation and decreased angiotensin 1–7 levels. Angiotensin 1–7 is now considered to be an important anti-inflammatory and anti-thrombotic peptide with inhibitory effects on platelet activation [14]. Hence, by binding to the ACE2 receptor, SARS-CoV-2 may induce renin–angiotensin axis dysregulation and coagulation disorders. Moreover, the overproduction of angiotensin II can trigger the activation of NADPH oxidase that may, in turn, enhance oxidative stress mechanisms and the release of inflammatory molecules, leading to the rapid progression of the disease [15]. It was postulated that, by this mechanism, the virus leads to the excessive release of different inflammatory cytokines, causing a disturbancein the the regulation of the renin–angiotensin–aldosterone system, an attenuation of Mas receptor (ACE2/MasR axis) and the activation of the complement system [16]. This “cytokine storm” is directly related to the acute symptoms and progression of inflammation [17].

TMPRSS2 expression, a cell surface protein primarily expressed by endothelial cells and involved in cleaving peptide bonds of proteins, may also explain the target cell types and clinical manifestation of SARS-CoV-2 infection [18]. TMPRSS2 expression is similar to ACE2 expression in many tissues, such as the kidney, liver, and testicles, and among the gastrointestinal tract, especially in the small intestine, as well as in the lungs, mainly in type II alveolar cells [19]. It was demonstrated that TMPRSS2 activates the spike protein domain (a key glycoprotein found on coronaviruses), which leads to the virus fusing to the respiratory epithelia on the cell surface through binding to ACE2. Hence, interactions between the spike-domain on viruses and TMPRSS2 are critical for viral entry into epithelial cells in the respiratory and digestive tract [20].

Interferon tissular levels seem to correlate with disease severity among COVID-19 patients [21]. Although interferon is a main contributing component against viral infections, it can also promote tissue injury when its level increases, suggesting that organ location could be related to IFN-mediated immune responses [22]. Neutrophil extracellular traps (NET), one of several critical components of innate immunity, can become dysregulated in patients infected with SARS-CoV-2 and were suggested to play a main role in SARS-CoV-2-related tissue injury [23]. Once activated, NETs promote a positive feedback loop that drives platelet aggregation and cytokine release, as well as complement activation, and is associated with microthrombosis [24,25]. Plasma, tracheal aspirates, lung tissue, and arterial thrombi from patients with severe COVID-19 all contain increased numbers of NETs, suggesting the role of these mediators in COVID-19-related epithelial and endothelial injury [26,27,28].

**Figure 2:**
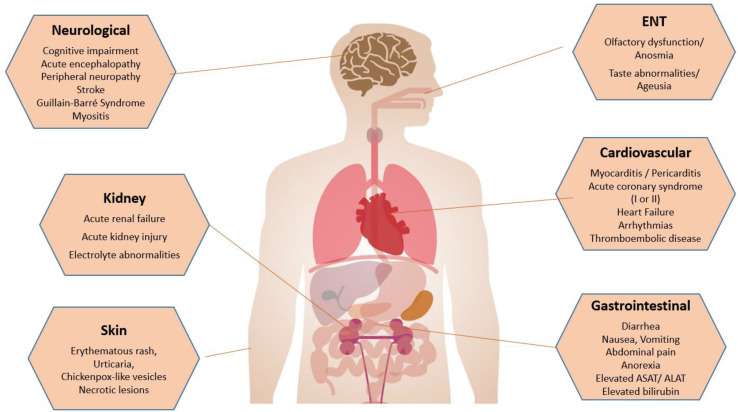
Summary of extrapulmonary manifestations of SARS-CoV-2 infection.

A summary of pathophysiological and molecular mechanisms of SARS-CoV-2 infection is shown in Figure 2.

## 3. Neurological Involvement

### 3.1. Mechanisms of Injury

Single-nucleus transcriptome analyses failed to detect viral transcripts in the neurons of patients with COVID-19 infection [29]. However, ACE2 is expressed in some areas of the brain [30], and some SARS-CoV-2-related neurologic symptoms, such as headache and encephalopathy, support the viral ability to reach the central nervous system. Baig et al. proposed virus dissemination via a haematological route or via the cribriform plate to bind to the epithelial ACE2 receptor [31]. Other mechanisms proposed for SARS-CoV-2-related neurological injury include increased cellular activation, mobility, and phagocytosis with perturbations in the choroid plexus [32], mainly affecting the synaptic signalling of excitatory neurons like changes seen in some neurodegenerative disorders.

### 3.2. Common Clinical Manifestations

Several neurological manifestations were described in COVID-19 infections, affecting both the central nervous system (CNS) and peripheral nervous system (PNS).

Anosmia is the most frequently described condition, affecting more than half of the patients [33]. SARS-CoV-2 could reach the CNS by infiltrating the olfactory neuroepithelium, which is rich in ACE2 receptors [34,35].

Headache is a common symptom during the acute phase of COVID-19 infection, affecting between 14 and 60% of patients [36,37]. This symptom frequently persists in the aftermath of the infection. Indeed, between 8 and 15% of patients have persistent headaches after a COVID-19 infection [38]. The frequency of the headaches seems to be similar in severe and non-severe cases of COVID-19 [38]. A persistent headache seems to be favoured by a genetic predisposition to migraine and is more frequent in patients with pre-existing headaches. Underlying mechanisms can include the activation of the trigeminovascular system, persistent activation of the immune system, or functional changes in grey and white matter [39].

Cognitive impairment was also frequently described after a COVID-19 infection. During the acute phase, the prevalence was 61.5% in a cohort of patients hospitalized for mild or moderate symptoms of COVID-19. A systematic review showed moderate cognitive impairment in more than half of cases [40]. This was found both in patients with moderate and severe COVID-19 [41]. Severe neurocognitive impairment after moderate-to-severe COVID-19 was estimated at 18.4% at 2 months after discharge [42]. Immediate verbal memory, semantic verbal fluency, attention, executive functions, and delayed memory seem to be the main cognitive domains impaired [40]. A meta-analysis of COVID-19 patients versus a control group showed a reduction of 0.94 points on the Montreal Cognitive Assessment in patients with COVID-19 infection (MD = −0.94, 95% CI—1.59, −0.29; *p* = 0.0049) [40].

### 3.3. Clinical Manifestations of Severe SARS-CoV-2

The spectrum of neurological complications in the severe COVID-19 infection is now well known thanks to several big cohorts from multiple countries [43,44]. The neurological manifestations of COVID-19 may be so severe that they can involve respiratory function, raising a therapeutic challenge between respiratory distress care and neurological manifestation management.

At the beginning of the COVID-19 pandemic, some limited articles reported neurological complications arising 2 to 3 weeks after the onset of COVID-19, associating axonal peripheral neuropathy or inflammatory myopathy in relation to inflammatory microangiopathy able to induce respiratory distress [44,45].

Then, in addition to these neuro-muscular syndromes, large-vessel ischemic strokes were reported related either to pre-existing co-morbidities such as atheroma, heart failure, or atrial fibrillation or related to inflammatory cerebral vasculitis or pro-thrombotic storm due to COVID-19 [43,46] Most comprehensive data about neurologic involvement in COVID-19 were derived from three large consortia analysing 3055 patients [44]. Overall, 80% of evaluated patients exhibited at least a new neurological symptom, sign, or syndrome. Acute encephalopathy was the most common clinically neurological syndrome (49%), with a reported incidence of coma at 17%. The direct invasion of SARS-CoV-2 into neuronal cells, or encephalopathy due to hypoxia, seizures, acute disseminated encephalomyelitis, or renal dysfunction, are the main postulated mechanisms [43] Stroke was reported in 3–6%, mainly of the ischemic type, which may have been induced by a pro-thrombotic storm (which could explain the association with a concurrent pulmonary embolism) or to COVID-19-related cerebral vasculitis, which could also explain haemorrhagic stroke [47]. Cerebral venous sinus thrombosis was reported in COVID-19, but this complication may also be observed after the Ad26.COV.2.S vaccination, which is associated with thrombocytopenia [48]. Intubation and mechanical ventilation were necessary in stroke patients with COVID-19 in 12.6% versus in 3.5% in stroke without COVID-19 [49].

The prevalence of Guillain–Barre syndrome (GBS) was estimated in a meta-analysis at 15 cases per 100,000 cases of COVID-19. This rate was higher among hospitalized patients, reaching 0.4% [50]. On average, signs of GBS appeared 19 days after the first symptoms of COVID-19. Signs reported are weakness (generalized in 67% of cases), hypoesthesia (42%), paraesthesia (27%), respiratory muscle involvement (18%), facial paralysis (35%), bulbar nerve involvement (17%), ophthalmoplegia (12%), and trigeminal hypoesthesia (6%). Concomitant CNS involvement was reported in a few cases, mainly with decreased levels of consciousness and delirium/confusion [51]. Albumin cytological dissociation was found in 75% of cases. In electroneuromyography, axonal damage was described in 42% of cases; the outcome of GBS associated with COVID-19 infection was often favourable; in 82% of cases, patients completely recovered or experienced an improvement of their symptoms [51].

Specific inflammatory myositis was reported in 23% of severe COVID-19 [43]. The prevalence of ICU-acquired weakness, also called ICU-related neuro-myopathy in COVID-19, was reported in 61–75% of patients needing mechanical ventilation [52,53]. This prevalence is higher than that reported in a large series of ventilated patients for other causes [54]. This syndrome is induced by injury to the nerves and muscles. It may arise during or after post-intensive care as the sum of several factors, such as hypoxia, cardiogenic shock, inflammatory storm, acute undernutrition, immobility, hypercatabolism, or sepsis [55].

### 3.4. Specific Considerations Regarding Preexisting Neurological Diseases

It is recommended that patients with neuromuscular disorders be monitored for the early identification of a decline in pulmonary function that requires ventilatory support [56].

Hydroxychloroquine, a drug used to treat COVID-19 infection, may worsen myasthenia gravis symptoms, and it is contraindicated in those patients [57].

## 4. Cardiovascular Manifestations

### 4.1. Mechanism of Injury

Microvascular injury is, nowadays, the main postulated mechanism explaining cardiac injury among patients with SARS-CoV-2 infection [58,59].

The underlying mechanisms postulated to explain this microvascular injury include NET formation and platelet activation promoting coronary thrombosis and endothelial damage [58,59]. The virus was not identified in cardiac muscle cells, keeping away the hypothesis of a direct action of SARS-CoV-2- through myocardial tissue. Some studies have shown a decrease in the number of cardiomyocytes and pericytes relative to endothelial cells in patients infected by SARS-CoV-2 [60].

Another mechanism suggested to explain COVID-19-related cardiovascular injury is the increase in vascular stiffness. Arterial stiffness is considered a major risk factor for cardiovascular disease development [61]. In this field, increases in arterial stiffness have been described during the acute phase of the SARS-CoV-2 infection [62] that can persist for up to one year [63]. As a dysfunctional endothelium led to a reduction in nitric oxide availability, leading to vasoconstriction, it has been hypothesized that one of the main mechanisms explaining the effects of SARS-CoV-2 infection on arterial stiffness is endothelial dysfunction [64]. However, increased stiffness can also induce further endothelial damage, resulting in a vicious cycle that can lead to long-term cardiovascular consequences in COVID-19 patients.

In conclusion, endothelial dysfunction and vascular stiffness seem to be the main causal factors for the initiation and progression of atherosclerotic disease by SARS-CoV-2 infection.

### 4.2. Clinical Manifestations

The spectrum of cardiac manifestations described in patients with COVID-19 is large and includes myocardial infarction with (type 1) or without (type 2) obstructive coronary artery disease, arterial or venous thromboembolic disease, pericarditis and myocarditis, cardiac arrhythmias, acute heart failure, and cardiogenic shock [65]. Although the clinical presentation of COVID-19 infection is dominated by respiratory symptoms, it is now well established that the virus can itself cause direct life-threatening cardiovascular injury [66], probably by direct action of SARS-CoV-2 on ACE2 receptors that are extensively expressed in the heart. Its impact is more severe in patients with preexisting cardiac disease or cardiovascular comorbidities [61].

Myocardial injury manifested by elevations in cardiac troponin (troponin elevation > 99th percentile) is common in patients with SARS-CoV-2 infection. Previous studies show that acute myocarditis or pericarditis is frequent among infected patients, especially in patients requiring intensive care [67,68,69]. In patients with COVID-19, viral myocarditis is an important cause of myocardial injury. It manifests with an “infarct-like” presentation consisting of elevated cardiac biomarkers and ECG changes in the absence of coronary artery disease [67]. Cardiac inflammatory involvement is probably due to the reduction of ACE2 levels, increase in angiotensin II relative to angiotensin 1–7, hypoxia, and/or abnormalities in the coagulation pathway. The inflammatory response plays a large role in myocarditis resulting from SARS-CoV-2 infection [70]. Indeed, acute myocarditis can lead to serious complications, such as sudden cardiac death or dilated cardiomyopathy with acute heart failure, and the disease was identified as a potential cause of death in the context of COVID-19 infection, mainly in young patients. It should also be mentioned that myocarditis/pericarditis is a rare complication of COVID-19 mRNA vaccinations, especially in young adult and adolescent males [71,72].

Recent studies suggest that SARS-CoV-2 infection increases the risk of acute coronary syndrome and, in particular, of acute MI [73]. Potential mechanisms for this increased risk include an exaggerated inflammatory response (cytokine storm) or a direct effect of the virus on endothelial cells through ACE2 receptor downregulation, platelet activation, and hypercoagulability that may destabilize coronary artery plaques [74]. Even if the majority of SARS-CoV-2-related MI are type 2 (consequent to a mismatch between oxygen supply and demand) and related to respiratory injury, hemodynamic instability, hypotension, and sepsis-related increases in myocardial oxygen consumption, a type 1 infarction (acute atherothrombotic coronary artery disease usually precipitated by atherosclerotic plaque disruption) is possible due to hypercoagulability associated with COVID-19 infection [75].

Moreover, some studies suggested a link between SARS-CoV-2-related pulmonary injury and the risk of acute myocardial infarction (MI), both of type 1 and type 2 [76]. Underlying mechanisms are only partially understood, but they could include coronary endothelial dysfunction, platelet activation leading to subsequent coronary thrombosis (type 1 MI), and sepsis-related increases in myocardial oxygen consumption in the absence of atherothrombotic events (type 2 MI).

Cardiac arrhythmias such as supraventricular and ventricular arrhythmias or conduction disorders are also commonly reported in COVID-19 [77,78]. Some studies showed that the development of arrhythmias was associated with higher mortality. Mechanisms such as hypoxia, electrolyte disorders, and the administration of arrhythmogenic medications (e.g., hydroxychloroquine, azithromycin) make it difficult to assert the direct and indirect contribution of COVID-19 on cardiac arrhythmias.

Acute heart failure with respiratory distress was found to be a possible consequence of COVID-19, with a dramatic impact on mortality [79]. During COVID-19 hospitalization, it was suggested that about one-third of patients with previous HF had an acute decompensation of HF [80]. However, acute HF can appear de novo and be linked to one of the conditions previously described. Echocardiographic findings reported in COVID-19 patients included acute HF with LV systolic dysfunction, RV systolic dysfunction, or LV diastolic dysfunction, according to the underlying condition.

## 5. Gastrointestinal and Liver Abnormalities

### 5.1. Mechanisms of Injury

SARS-CoV-2 can infect and replicate in the gastrointestinal tract and is a potential transmission route for SARS-CoV-2, which could have serious consequences for disease control and transmission [81]. Gastrointestinal symptoms may be due to direct aggression, an alteration of the ACE2 function, or a secondary effect of interference in the balance of the gastrointestinal tract due to the change in the intestinal microbiota. The negative regulation of ACE2 by the virus also may reduce the production of metabolites implicated in the regulation of intestinal homeostasis [82]. Finally, gastrointestinal injury may be associated with damage to the intestinal epithelium due to the effects of the cytokine storm [83]. Hepatic changes can be caused by direct damage to cholangiocytes and hepatocytes, as well as the consequence of a cytokine storm [84]. The so-called gut–lung axis is another mechanism explaining intestinal symptoms during COVID-19: hypoxemia can cause changes in the intestinal microbiota and tissue damage [82].

### 5.2. Clinical Manifestations

During SARS-CoV-2 infections, GI symptoms may precede respiratory symptoms but also can be the only symptoms of the disease [85]. The prevalence of these GI symptoms was reported to vary between 3 and 79% in different series [86].

The most common symptoms were loss of appetite, nausea, and vomiting, which occurred in about 67–84% of the patients, diarrhoea occurred in 29–37%, and abdominal pain occurred in 4–25% [87,88].

Interestingly, patients with gastrointestinal symptoms were more likely to have a more severe disease characterized by greater degrees of liver insult, coagulopathy, the development of ARDS, ICU admission, and the necessity for more frequent mechanical ventilation [88,89]. The pathophysiology of GI insult is probably multifactorial. Virus-mediated direct tissue injury is possible, given the presence of ACE2 in intestinal cells, as well as the visualization of viral proteins in gastrointestinal cells [90].

It was described that ACE2 expression is higher in the epithelial cells of the colon of patients with adenomas or colorectal cancer than in healthy adults, which may suggest a higher risk of infection with SARS-CoV-2 in those patients [91,92].

ACE2 is expressed in cholangiocytes and could be responsible for direct cytotoxicity [93]. Reported liver abnormalities related to COVID-19 infection include elevations in serum levels of alanine–transaminase (ALAT), aspartate–transaminase (ASAT), and bilirubin. A recent systematic review reported a pooled prevalence of liver function abnormalities at 19% [94].

It was suggested that the degree of liver injury may be associated with disease severity. A recent meta-analysis showed that the presence of higher levels of ALAT (1.5–1.8-fold), ASAT (1.8-fold), and total bilirubin (1.2–1.3-fold) are related to a higher rate of ICU admissions [95].

## 6. Other Manifestations of COVID-19 Infection

Patients with diabetes mellitus and/or obesity are at risk of developing more severe COVID-19 disease. The odds of severe illness and death risk were estimated to be up to twofold greater in patients with diabetes compared to those without the condition [96]. Moreover, glucose control seems to impact COVID-19 infection prognosis in diabetic patients. Hence, patients with uncontrolled diabetes have, respectively, a twofold increase and five times increase in rate of hospitalization and ICU admission than those with good glycaemic control [97]. However, the number of studies comparing the prognosis of type 1 and type 2 diabetes is limited, making it difficult to estimate the differential risk of both conditions [98]. In addition, a range of endocrinology manifestations, including hyperglycaemia, ketoacidosis in patients with previously undiagnosed diabetes, and non-diabetic euglycemic ketosis, were described in patients with COVID-19 infection [99]. Mechanisms evoked to explain these findings include impairment in pancreatic β-cell function secondary to elevated cytokine levels and direct binding of SARS-CoV-2 to ACE2 on β-cells [100,101].

Few data are available about the impact of obesity on COVID-19 patients. However, some studies suggested that obesity may increase the susceptibility to severe ARDS, respiratory failure, and an increase in mortality [102].

Acute kidney injury in patients with COVID-19 was reported in 5 to 37%, according to a published series [85,103,104]. Most common clinical presentations include electrolyte abnormalities (hyperkalaemia, hyponatremia, and hypernatremia, among others), haematuria, proteinuria, and metabolic acidosis.

Several mechanisms were suggested to explain renal injury in COVID-19. SARS-CoV-2 has a significant affinity for the ACE-2 that is highly expressed in the kidney and could be a target for direct toxicity. Viral inclusion particles were visualized by electron microscopy in the tubular epithelium and glomerular capillary loops [105]. Moreover, SARS-CoV-2 induces the release of cytokines IL-2, IL-7, and IL-10, which are believed to be potentially involved in the pathology of kidney injury [106]. Finally, in severe forms of the disease, hypovolemia, rhabdomyolysis, hypoxemia, and septic shock may crucially contribute to renal impairment.

Nasal epithelial cells display the highest expression of ACE2 in the respiratory system [107]. This may explain why smell and/or taste abnormalities were frequently reported in COVID-19 patients. A European multicentre study showed that 85.6% and 88.0% of patients reported olfactory and gustatory dysfunctions, with an early recovery rate of 44%. Olfactory dysfunction (OD) appeared before the other symptoms in 12% of patients [108]. The authors suggest that sudden anosmia or ageusia need to be recognized as important markers of the COVID-19 infection. Moreover, as taste and olfactory changes are an early manifestation of the disease, they can serve as a screening tool for identifying people with SARS-CoV-2 infection and limit viral spread [109].

Cutaneous manifestations are rare in SARS-CoV-2 infection, with reported rates of less than 2%. Nonspecific dermatologic manifestations were reported, mostly erythematous rash, urticaria, chickenpox-like vesicles, livedoid, and necrotic lesions [110,111]. These patterns of injury likely reflect cutaneous reactions to circulating viral antigens and, thus, typically feature endothelial injury and perivascular inflammation of dermal vessels. Nearly 50% of patients with rashes related to SARS-CoV-2 have erythematous or maculopapular eruptions that develop after the onset of systemic symptoms. These eruptions seem to reflect some type of vascular injury, often with perivascular lymphocytic or neutrophilic infiltrates [112,113].

The impact of drugs such as hydroxychloroquine, remdesivir, tocilizumab, and other experimental drugs should always be eliminated before attributing skin lesions to the viral infection.

A summary of extrapulmonary manifestations of SARS-CoV-2 infection can be seen in Figure 2.

## 7. Perspectives

Future perspectives and challenges regarding SARS-CoV-2 infection could focus on a better understanding of the underlying pathophysiological mechanism of the disease, on the evaluation of the impact of the emergence of viral resistance strains and new variants, on the definition of the role of preventive measures and vaccines, and finally, on the development of innovative treatments targeting the viral and inflammatory phase.

## 8. Conclusions

While the main clinical manifestations of COVID-19 are related to the SARS2-COV-2 pulmonary injury, extrapulmonary manifestations of COVID-19 are frequent and can affect multiple organs, including cardiovascular, neurological, gastrointestinal, renal, hepatic, and dermatologic systems. Therefore, SARS-CoV-2 infection is a multifactorial process and involves the combined effects on structures, enzymes, and immune responses. The pathological manifestations and damage caused are manifested as a process in which the lung is the main object of damage, but it also causes extensive extrapulmonary injury as the disease progresses. Clinicians of different specialties should be aware of the potentially multiorganic impact of COVID-19 and its influence on the disease course and prognosis, as this can have a relevant influence on their daily clinical practice.
